# Identification and characterization of nanobodies specific for the human ubiquitin–like ISG15 protein

**DOI:** 10.1016/j.jbc.2025.110564

**Published:** 2025-08-06

**Authors:** Jin Gan, Prachi Dabhade, Charlotte Wijne, William McKibben, Simeon D. Draganov, Hedib Alrawili, Zhen-Yu Jim Sun, Jack W. Houghton, Edward W. Tate, Camille Le Gall, Pavana Suresh, Novalia Pishesha, Adán Pinto-Fernández, Thomas U. Schwartz, Hidde L. Ploegh

**Affiliations:** 1 Program in Cellular and Molecular Medicine, Boston Children's Hospital, Harvard Medical School, Boston, Massachusetts, USA; 2 Department of Biology, Massachusetts Institute of Technology, Cambridge, Massachusetts, USA; 3 Department of Cell and Chemical Biology, Leiden University Medical Center, Leiden, The Netherlands; 4 Translational Ubiquitomics Laboratory, Nuffield Department of Medicine, CAMS Oxford Institute and Centre for Medicines Discovery, University of Oxford, Oxford, United Kingdom; 5 Department of Cancer Biology, Dana-Farber Cancer Institute, Boston, Massachusetts, USA; 6 Department of Chemistry, Molecular Sciences Research Hub, Imperial College London, London, United Kingdom; 7 Division of Immunology, Boston Children’s Hospital, Harvard Medical School, Boston, Massachusetts, USA

**Keywords:** nanobody, VHH, ISG15, protein structure, NMR, USP16

## Abstract

Interferon-induced ubiquitin (Ub)-like modifier Interferon Stimulated Gene 15 (ISG15) functions both intracellularly and as a secreted protein with cytokine-like properties. The ISG15 pathway is implicated in various diseases, including cancer and inflammatory disorders, but understanding its precise roles has been challenging because of limited availability of tools to study ISG15 biology. Here, we report the development of two novel nanobodies that target human ISG15, obtained through alpaca immunization and phage display. These nanobodies, VHH_ISG15-A_ and VHH_ISG15-B_, exhibit nanomolar binding affinities and recognize distinct epitopes on ISG15's C- and N-terminal domains, respectively, as demonstrated by NMR and X-ray structural analyses. Both nanobodies enable the immunoprecipitation and proteomic identification of ISGylated substrates with minimal background contamination. VHH_ISG15-A_ is compatible with immunoblotting and recognizes unconjugated ISG15 under denaturing conditions. Functional assays showed that VHH_ISG15-A_, but not VHH_ISG15-B_, inhibits ubiquitin-specific peptidase 16–mediated deISGylation, likely by steric hindrance at the ISG15-binding interface. These results underscore the utility of VHH_ISG15-A_ and VHH_ISG15-B_ as tools to study ISG15 biology.

Ubiquitin (Ub) and Ub-like modifiers control many aspects of cellular physiology (1). The Interferon Stimulated Gene 15 (ISG15) encodes a Ub-like modifier with a molecular weight of approximately 15 kDa (2). As suggested by its name, the expression of the ISG15 gene is modulated by exposure to interferon (IFN). ISG15 is unusual in that it exerts its function both intracellularly and as a secreted protein with cytokine-like properties to stimulate IFN-γ secretion (3, 4). Free, intracellular ISG15 can affect protein–protein interactions (5, 6, 7). In humans, unconjugated ISG15 can bind to and stabilize ubiquitin-specific peptidase 18 (USP18), a key negative regulator of IFN signaling. This interaction spares USP18 from degradation, leading to increased USP18 levels. Consequently, patients deficient in ISG15 show enhanced IFN-α/β immunity, which can result in interferonopathies and necrotizing skin lesions (7, 8). Beyond this regulatory role, ISG15 is also widely recognized for its involvement in the host's innate immune response to viral infections through the modification of both viral and host proteins (9). ISG15 requires activation for conjugation to a suitable substrate. The activation of ISG15 follows a path similar to that of the activation of Ub. Prior to conjugation, the ISG15 precursor protein must be processed by deISGylating enzymes to reveal its carboxy-terminal LRLRGG motif (10). An E1-type activity, encoded by UBE1L (11), generates an E1 thioester–linked version of ISG15. Transfer of ISG15 to substrates requires the action of a single E2-type activity, encoded by UBE2L6/UbcH8 (12). ISG15 is then transferred to substrate(s) in an E3-dependent manner, primarily by HERC5 (13), a process referred to as ISGylation. All components of the ISG15 activation pathway are themselves induced upon exposure of cells to IFN. ISGylation is reversible: USP18 (14), USP21 (15), USP16 (16), and USP24 (17) have all been reported to remove ISG15 from ISG15-modified substrates.

Changes in the ISG15 pathway are associated with a number of diseases, including cancer, neurodegenerative disorders, infection, and inflammation (18). However, the consequences of the ISGylation–deISGylation cycle are complex and less well understood than the diverse roles of ubiquitylation. This knowledge gap stems primarily from the low abundance of ISGylated substrates in cells and the limited characterization of the ISG15 substrate repertoire. Mass spectrometry (MS)–based proteomics coupled with ISG15-specific purification strategies, such as antibody-mediated capture of endogenous ISG15 conjugates (19, 20), or affinity-based pulldown using tagged ISG15 expression systems (21, 22, 23, 24) have sought to address this question. Besides these, anti-K-ε-GG antibodies, which enable proteome-wide mapping of ISG15 (and Ub) substrates and their specific modification sites (25, 26), have been used.

Here, we undertook the generation of new tools to study the role of ISG15. We immunized alpacas with recombinant human ISG15 to obtain ISG15-specific nanobodies. Nanobodies are the recombinantly expressed variable regions of the heavy chain–only immunoglobulins found in camelids. Their single domain nature allows nanobodies to probe epitopes not always accessible to conventional immunoglobulins. Moreover, many nanobodies retain their antigen-binding properties when expressed in the reducing environment of the cytosol. Additional benefits of nanobodies include superior tissue penetration, high-yield bacterial production, and ease of modification (27, 28).

We obtained two ISG15-specific nanobodies with nanomolar affinity by phage display of a library constructed from lymphocytes of an alpaca immunized with ISG15: VHH_ISG15-A_ and VHH_ISG15-B_. We confirmed their distinct binding sites, with VHH_ISG15-A_ targeting the C-terminal Ub-like domain and VHH_ISG15-B_ recognizing the N-terminal domain. Both nanobodies, when immobilized, readily recovered ISGylated substrates. This enabled their proteomic identification. Functional assays showed that VHH_ISG15-A_ inhibits USP16-mediated ISG15 processing, whereas VHH_ISG15-B_ does not. These tools not only allow mechanistic studies of ISG15 but may also intercept processes driven by dysregulation of the pathways that involve ISG15.

## Results

### Identification of human ISG15–specific nanobodies

**Figure 1:**
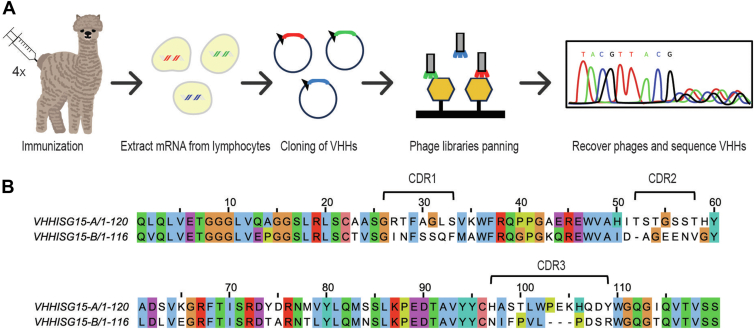
**Identification of human ISG15–specific nanobodies.***A*, scheme of alpaca immunization and ISG15 nanobody (VHH) selection by phage display. *B*, sequence alignment of the identified ISG15 nanobodies, with indication of the complementarity determining regions (CDRs). ISG15, Interferon Stimulated Gene 15.

To generate human ISG15–specific nanobodies, we immunized an alpaca (*Vicugna pacos*) with recombinantly expressed, purified full-length human ISG15. Induction of an immune response was assessed by immunoblotting, using sera from the immunized animal after the third boost. Having detected a specific signal with immune serum, but not with preimmune serum or secondary antibody only, we harvested ∼50 ml of peripheral blood and isolated mononuclear cells. RNA was prepared and used as a template for PCR amplification of VHH-specific sequences using VHH-specific primers (29). The resulting complementary DNAs (cDNAs) were cloned into a phage display vector to obtain a library suitable for panning against ISG15, using standard procedures (Fig. 1*A*).

Panning was performed using immobilized human ISG15 as the bait protein. Sequencing of 96 individual nanobody clones isolated after two subsequent rounds of panning identified 20 closely related VHH sequences as well as a number of unique sequences. We refer to the ISG15-specific VHHs as VHH_ISG15-A_ and VHH_ISG15-B_ (Fig. 1*B*) based on their detailed characterization described in the following. These VHHs use different germline V segments and have widely divergent sequences for CDR3 in particular, already suggesting that they recognize distinct epitopes on ISG15.

We subcloned VHH_ISG15-A_ and VHH_ISG15-B_ sequences into the pHEN6 periplasmic expression vector for large-scale production in *Escherichia coli*. We also installed a C-terminal LPETGG-HA-His6 tag to facilitate protein purification, detection, and allow the nanobody to participate as a substrate in a sortase-based transpeptidation reaction. Both VHH_ISG15-A_ and VHH_ISG15-B_ were produced and purified in good yield (∼32 mg/l of culture and ∼34 mg/l of culture for VHH_ISG15-A_ and VHH_ISG15-B,_ respectively).

### Binding validation of ISG15-specific nanobodies

**Figure 2:**
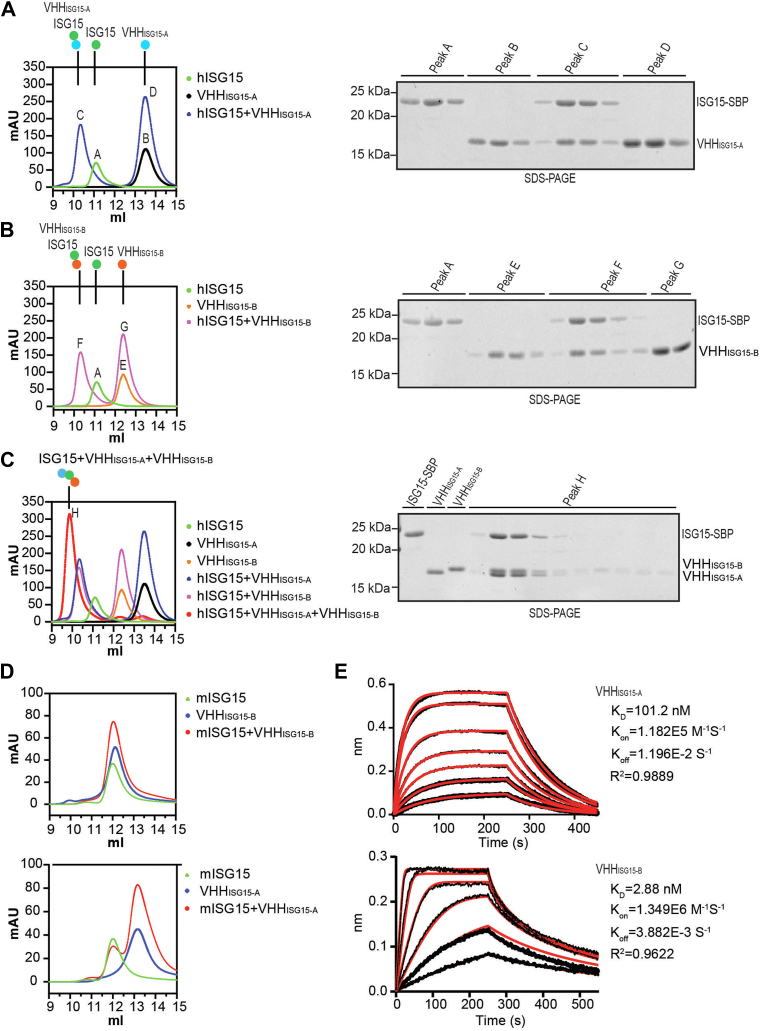
**Binding validation of ISG15-specific nanobodies.***A* and *C*, binding of nanobodies to hISG15-SBP-His as determined by size-exclusion chromatography (SEC). *A*, profiles of hISG15-SBP-His (*green*, peak A), VHH_ISG15-A_ (*black*, peak B), and a 1:2 M ratio mixture of hISG15-SBP-His:VHH_ISG15-A_ (*blue*, peaks C and D) are shown. *B,* profiles of hISG15-SBP-His (*green*, peak A), VHH_ISG15-B_ (*orange*, peak E), and a 1:2 M ratio mixture of hISG15-SBP-His:VHH_ISG15-B_ (*pink*, peaks F and G) are shown. *C*, profile of a 1:1:1 M ratio mixture of hISG15-SBP-His:VHH_ISG15-A_:VHH_ISG15-B_ (*red*, peak H) is shown. SDS-PAGE gels of the 10 to 14 ml fractions corresponding to the VHH:ISG15–SBP complex separated by SEC are shown on the *right panel*. *D*, binding of VHHs to His-mISG15 as determined by SEC. Profiles of His-mISG15 (*green*), VHH (*blue*), and a 1:2 M ratio mixture of His-mISG15:VHH (*red*) are shown. *E*, representative sensorgrams from biolayer interferometry (BLI) analysis showing the binding interactions of ISG15 with VHH_ISG15-A_ (*top panel*) and VHH_ISG15-B_ (*bottom panel*), respectively. Experimental association and dissociation data (*dots*) are overlaid with globally fitted binding model curves (*solid red lines*). The data (n = 3) were used to calculate the equilibrium dissociation constant (*K*_*D*_), association rate constant (*K*_on_), and dissociation rate constant (*K*_off_). ISG15, Interferon Stimulated Gene 15; SBP, streptavidin-binding peptide.

To confirm the interaction between human ISG15 and newly identified VHHs, we mixed a twofold molar excess of each of the individual VHHs with ISG15 and analyzed these mixtures by size-exclusion chromatography (SEC) on a Superdex S75 column and compared the elution profiles with those of the individual components (Fig. 2, *A* and *B*). Both VHH_ISG15-A_ and VHH_ISG15-B_ bind to ISG15 in solution, as evident from the appearance of a novel peak when each of the nanobodies is combined with ISG15. To determine whether VHH_ISG15-A_ and VHH_ISG15-B_ could bind to ISG15 simultaneously, we prepared a 1:1:1 mixture of hISG15-SBP (streptavidin-binding peptide)-His:VHH_ISG15-A_:VHH_ISG15-B_ (Fig. 2*C*). The result unambiguously shows that both VHHs can bind to ISG15 independently. We also analyzed the mixtures of mouse ISG15 and VHHs by SEC, where no stable protein complex formation was observed (Fig. 2*D*). Therefore—and notwithstanding its structural similarity—mouse ISG15 does not interact with either VHH_ISG15-A_ or VHH_ISG15-B_. This is not unexpected in view of the limited sequence similarity between human and mouse ISG15.

Next, we performed biolayer interferometry (BLI) to analyze binding kinetics. We immobilized VHHs and used mutant human ISG15 (C78S) as the analyte to measure association and dissociation (Fig. 2*E*). Recombinant WT ISG15 is inherently aggregation prone because of disulfide bond–mediated dimerization through Cys^78^. A point mutation of cysteine 78 to serine markedly improves the accuracy of the BLI readout (30). VHH_ISG15-B_ (*K*_*D*_ = 2.88 nM) has an affinity more than 35-fold higher than VHH_ISG15-A_ (*K*_*D*_ = 101.2 nM).

### Solution NMR analysis of the ISG15–VHH complexes

**Figure 3:**
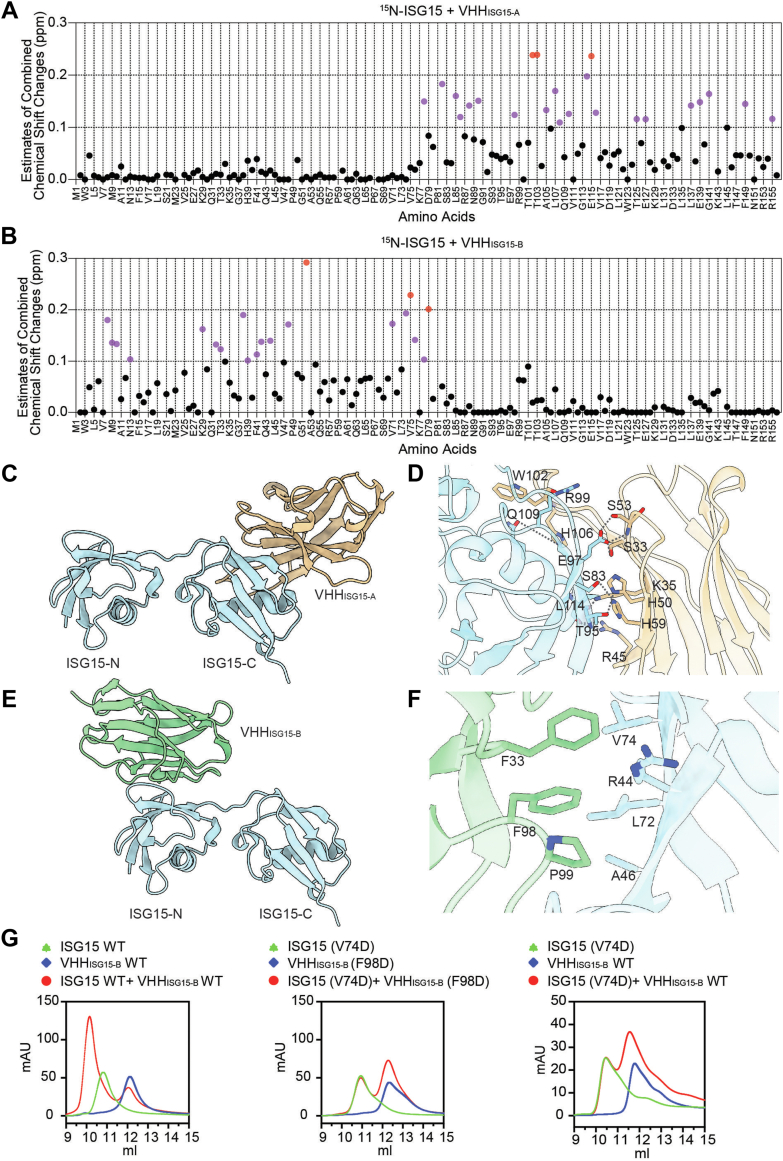
**Binding characterization of ISG15-specific VHHs.***A* and *B*, estimated combined 1H/15N-chemical shift changes in ppm of 15N-labeled human ISG15 in the presence of excess VHH_ISG15-A_ (*A*) and VHH_ISG15-B_ (*B*). Individual ISG15 residues with chemical shift changes >0.1 ppm are indicated in *purple*, and those >0.2 ppm are indicated in *red*. *C*, crystal structure of VHH_ISG15-A_ in complex with human ISG15, resolved to 2.6 Å. *Cartoon representation* of ISG15 (*blue*) interacting with VHH_ISG15-A_ (*tan*) *via* the C-terminal domain. *D*, close-up view of the binding interface between ISG15 and VHH_ISG15-A_. Key interface residues are shown in *stick*. *E*, structure of VHH_ISG15-B_ in complex with human ISG15. *Cartoon representation* of AlphaFold3 predicted complex of ISG15 (*blue*) and VHH_ISG15-B_ (*green*) bound *via* the N-terminal domain. *F*, close-up view of the predicted binding interface between ISG15 and VHH_ISG15-B_. Key interface residues are shown in *stick*, H-bonds as *dashed lines*. *G*, SEC profiles depicting human ISG15 and VHH_ISG15-B_ binding. *Left panel,* ISG15 WT preincubated with VHHISG15-B WT at a 1:1 M ratio. *Middle panel,* ISG15 (V74D) preincubated with VHHISG15-B (F98D) at a 1:1 M ratio. *Right panel,* ISG15 (V74D) preincubated with VHHISG15-B WT at a 1:1 M ratio. ISG15, Interferon Stimulated Gene 15; SEC, size-exclusion chromatography.

The NMR assignments for ISG15 have been published (Biological Magnetic Resonance Data Bank entry: 5658) (31), allowing us to examine perturbations in chemical shifts upon incubation of each of the VHHs with ^15^N-labeled ISG15. We observed numerous differences when comparing the ^15^N-spectrum of free ISG15 with the spectra of ISG15 in the presence of VHH_ISG15-A_ or VHH_ISG15-B_. We tabulated the magnitude of the chemical shift for each residue that could be assigned unambiguously or estimated minimum shifts to nearest peaks and plotted these values against the amino acid sequence of ISG15. The results are clear: VHH_ISG15-A_ binds to the C-terminal Ub-like domain of ISG15 (Fig. 3*A*), whereas VHH_ISG15-B_ binds to the N-terminal Ub-like domain (Fig. 3*B*). The NMR data are thus fully consistent with the results from the SEC experiment. VHH-ISG15_A_ and VHH-ISG15_B_ recognize distinct, nonoverlapping epitopes and can bind to ISG15 simultaneously.

### Binding characterization of ISG15-specific nanobodies

To elucidate the molecular mechanisms underlying ISG15 recognition by the VHHs, we pursued experimental structural characterization of ISG15 in complex with VHH_ISG15-A_ and VHH_ISG15-B_ using X-ray crystallography. Despite numerous attempts, only the ISG15–VHH_ISG15-A_ complex yielded diffraction-quality crystals, enabling determination of its structure at 2.6 Å resolution (Table 1). The asymmetric unit contains three independent ISG15–VHH_ISG15-A_ complexes, which are practically identical (RMSD = 0.6–0.8 Å between complexes, Fig. S1). The crystal structure shows that VHH_ISG15-A_ establishes extensive contacts with the C-terminal Ub-like domain of ISG15, whereas the N-terminal domain remains structurally independent (Fig. 3*C*).

**Table 1:** Data collection and refinement statistics

Data collection	
Space group	*P* 2_1_
Cell dimensions	
*a*, *b*, *c* (Å)	68.4 114.4 79.3
*α*, *β*, *γ* (°)	90 105.8 90
Resolution (Å)	76.2 - 2.6 (2.7 - 2.6)
Total reflections	125,227 (7185)
Unique reflections	36,396 (2168)
Multiplicity	3.4 (3.3)
Completeness (%)	99.3 (95.6)
Mean I/sigma(I)	6.9 (1.2)
*R*-merge	0.13 (0.82)
*R*-pim	0.08 (0.52)
CC1/2	0.99 (0.34)
Refinement	
Resolution (Å)	76.2 - 2.6
No. of reflections	36,382 (2200)
No. of free reflections	2272 (138)
*R*-work	0.18 (0.28)
*R*-free	0.24 (0.32)
Number of nonhydrogen atoms	6505
Number of macromolecules	6330
Number of ligands	17
Number of solvents	158
RMS (bonds)	0.009
RMS (angles)	1.05
Ramachandran favored (%)	96.8
Ramachandran allowed (%)	3.1
Ramachandran outliers (%)	0.1
Average *B*-factor	
No. of macromolecules	57.7
No. of ligands	62.7
No. of solvents	50.2

Values in parentheses are for the highest-resolution shell (1/16th of the data).

Structural analysis of the binding interface showed key molecular interactions between ISG15 and VHH_ISG15-A_, stabilized by a hydrogen bond network (Fig. 3*D*). Within the CDR3 loop of VHH_ISG15-A_, L101 and W102 interact with R99 and H106 of ISG15. Additional stabilization is provided by interactions involving S33 and K35 of CDR1, as well as R45, H50, T52, and H59 of CDR2, which engage with S83, T95, E97, and L114 of ISG15, reinforcing the binding interface. These structural observations correlate well with the chemical shift perturbations observed in NMR analyses, particularly the pronounced shifts in the C-terminal domain.

We used AlphaFold3 (AF3) to predict the structure of the ISG15–VHH_ISG15-B_ complex, which was refractory to crystallization. The prediction had a high interface predicted template modeling score of 0.85 (32). The AF3 model suggests that VHH_ISG15-B_ exclusively engages the N-terminal domain of ISG15 (Fig. 3*E*), consistent with the observed NMR chemical shift patterns (Fig. 3*B*). The binding interface exhibits both hydrophobic and electrostatic complementarity, with L72 and V74 of ISG15 forming critical contacts within a hydrophobic pocket of VHH_ISG15-B_ created through F33 of CDR1 and F98/P99 of CDR3 (Fig. 3*F*). Additional stabilizing interactions are mediated by numerous ISG15 residues (Fig. S2). The predicted interactions were validated through site-directed mutagenesis. For this analysis, we picked hydrophobic residues V74 of ISG15 and F98 of VHH_ISG15-B_, both solvent exposed in the unbound state, but entirely buried upon binding. Point mutation V74D in ISG15 completely abrogated complex formation with either VHH_ISG15-B_ WT or VHH_ISG15-B_ (F98D), in agreement with the AF3 model (Fig. 3*G*).

To understand why our nanobodies, VHH_ISG15-A_ and VHH_ISG15-B_, bind specifically to human ISG15 but not mouse ISG15, we aligned the sequences of human and mouse ISG15. This alignment focused on the regions where the nanobodies bind (Fig. S2). The analysis revealed that the binding sites are not well conserved between the two species. Specifically, within the VHH_ISG15-A_ binding site, only 8 of 15 interacting residues are identical between human and mouse ISG15. For the VHH_ISG15-B_ binding site, only 7 of 16 interacting residues are conserved, and an additional two residues present in human ISG15 are entirely absent in the mouse sequence. This notable lack of conservation at the binding interfaces sufficiently explains the observed species-specific binding of both VHHISG15-A and VHHISG15-B to human ISG15.

Collectively, the integrated NMR and structural analyses show that VHH_ISG15-A_ and VHH_ISG15-B_ recognize distinct, nonoverlapping epitopes on ISG15. This structural complementarity explains their capacity for simultaneous binding.

### Biochemical characterization of ISG15-specific VHHs

**Figure 4:**
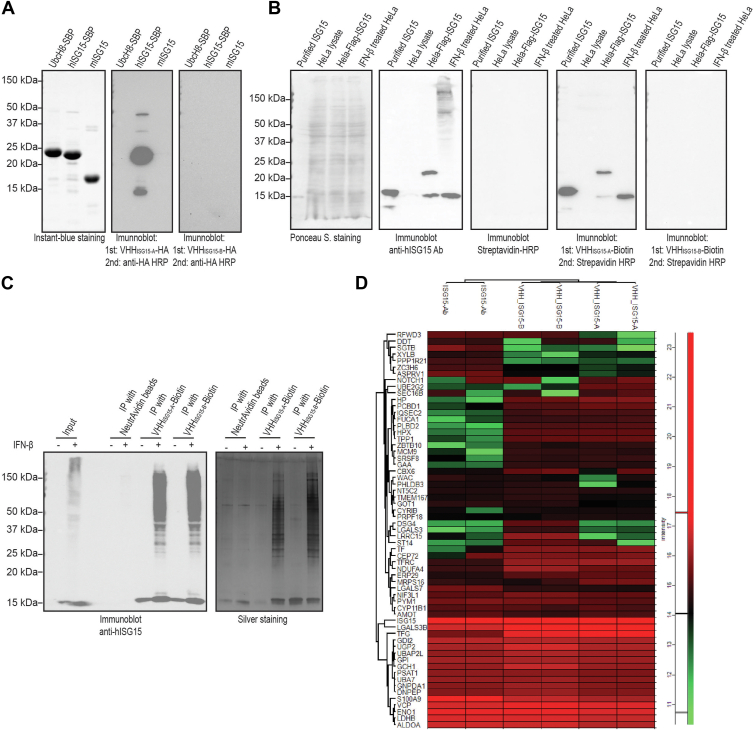
**Biochemical characterization of ISG15-specific VHHs.***A*, VHH_ISG15-A_, but not VHH_ISG15-B_, recognizes recombinant human ISG15 in immunoblot analysis. Recombinant hUbcH8-SBP-His (human), hISG15-SBP-His (human), and His-mISG15 (murine) proteins were separated by SDS-PAGE (*left*) and immunoblots (*right*) were performed with VHHs as the primary probes, followed by anti-HA monoclonal antibody to detect HA-tagged VHHs. Representative data of three (n = 3) independent experiments. *B*, VHH_ISG15-A_, but not VHH_ISG15-B_, detects cellular unconjugated ISG15 in HeLa cell lysates *via* immunoblot. HeLa-FLAG-ISG15:HeLa cell lysates exogenously expressing Tetra-FLAG–tagged ISG15. Representative data of three (n = 3) independent experiments. *C*, immunoprecipitation of ISGylated substrates using C-terminally biotin-tagged VHH_ISG15-A_ and VHH_ISG15-B_. Biotin-tagged VHH_ISG15-A_ and VHH_ISG15-B_ were prebound to streptavidin beads, followed by incubation with 1 mg of IFN-β-stimulated HeLa cell lysates for immunoprecipitation. Immunoprecipitated proteins were eluted by boiling, separated by SDS-PAGE, and detected by immunoblotting with anti-human ISG15 antibody (*left*) and silver staining (*right*). Representative data of three (n = 2) independent experiments. *D*, heatmap displaying the intensities of the top 30 interactors of VHH_ISG15-A_ (VHH-A) and VHH_ISG15-B_ (VHH-B) and an ISG15 antibody (ISG15-Ab), identified by LC–MS/MS analysis in HAP1 USP18 KO cells treated with IFN-α (500 U/ml) for 48 h. This experiment was performed in technical duplicates, the enrichment background was corrected using a beads-only control, and the top 30 interactors were selected based on Manhattan distances. IFN, interferon; ISG15, Interferon Stimulated Gene 15; USP18, ubiquitin-specific peptidase 18.

With two ISG15-specific VHHs in hand, we explored whether they are compatible with immunoblotting and immunoprecipitation (IP). We first performed immunoblotting against purified recombinant human UbcH8-SBP, human ISG15-SBP, and mouse ISG15 proteins. Blots were probed with 1 μg/ml C-terminally HA-tagged VHH_ISG15-A_ or VHH_ISG15-B_. Detection with anti-HA horseradish peroxidase (HRP) showed a clear signal for VHH_ISG15-A_ but not for VHH_ISG15-B_ (Fig. 4*A*). VHH_ISG15-A_ thus recognizes a feature on denatured human ISG15 when applied under reducing SDS-PAGE conditions. The specificity of VHH_ISG15-A_ was assessed by immunoblotting on lysates form HeLa cells stimulated with IFN-β. We used a sortase-based transpeptidation reaction to replace the C-terminal HA tag with biotin (33) and used streptavidin–HRP for detection. Only VHH_ISG15-A_ yielded an ISG15-specific signal with no obvious crossreaction with mammalian proteins. In contrast, no ISGylated protein bands were detected in the immunoblotting using VHH_ISG15-A_, unlike the ISG15 recombinant rabbit monoclonal antibody (clone 7H29L24) used in this study (Fig. 4*B*).

To evaluate whether ISG15-specific nanobodies can retrieve ISG15 from cell lysates, we performed IP on lysates from IFN-β-treated HeLa cells. The C-terminally biotin-tagged VHH_ISG15-A_ and VHH_ISG15-B_ were first preincubated with NeutrAvidin Agarose beads, then incubated with cell lysates from IFN-β-treated HeLa cells overnight. The recovered materials were resolved by SDS-PAGE and visualized by silver staining (Fig. 4*C*). Both VHH_ISG15-A_ and VHH_ISG15-B_ immunoprecipitated ISGylated proteins from the cell lysates, with minimal background contamination compared with the control with neutravidin beads and no VHH.

To assess the efficiency of IP using the two nanobodies more extensively, we designed label-free IP–MS experiments, with the inclusion of a recombinant rabbit monoclonal anti-ISG15 antibody for reference purposes (Table S1). The results showed a significant enrichment for ISG15 and, after hierarchical clustering, a good overlap between the two nanobodies and the ISG15 monoclonal antibody. Of note, each of the three enrichments detects a subset of proteins that are unique, in line with our clustering analysis (Fig. 4*D*) and consistent with the recognition of distinct epitopes. At least 17 protein hits can be found in previously published ISGylated substrate list (26); ALDOA and GOT1 have been validated in our previous study (16).

### VHH_ISG15-A_, but not VHH_ISG15-B_, inhibits USP16-mediated deISGylation *in vitro*

**Figure 5:**
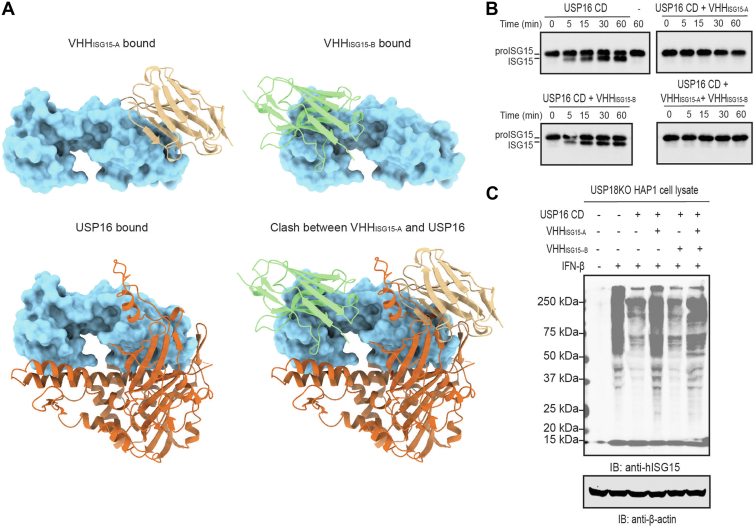
**VHH_ISG15-A_, not VHH_ISG15-B_, inhibits USP16-mediated deISGylation *in vitro*.***A*, *surface/cartoon representation* of ISG15 interacting with VHH_ISG15-A_, VHH_ISG15-B_ (AF3 predicted), and USP16 (AF3 predicted). *Top left,* VHH_ISG15-A_ (*tan*) interacts with ISG15 (*blue*) *via* the C-terminal domain. *Top right,* VHH_ISG15-B_ (*green*) interacts with ISG15 *via* the C-terminal domain. *Bottom left,* complex of ISG15 with USP16 (*brown*) predicted with AF3 interacts with ISG15 at the N- and C-terminal domains. For clarity, residues 392 to 604 of USP16 are not shown here as AF3 predicts it to be disordered. *Bottom right,* overlay of the three complex structures, aligned on ISG15. Steric clash between VHH_ISG15-A_ and USP16 is clearly apparent. *B*, cleavage of recombinant human proISG15 proteins by recombinant human USP16 in the presence and absence of a twofold molar excess of VHHs over proISG15 at the indicated time points. Proteins were separated by SDS-PAGE and immunoblotted with anti-human ISG15 antibody. Representative data of three (n = 3) independent experiments. *C*, deconjugation of ISGylated substrates by recombinant human USP16 in the presence and absence of 10 μM VHHs. Proteins were separated by SDS-PAGE and immunoblotted with anti-human ISG15 antibody. Probing for β-actin served as a loading control. Representative data of two (n = 2) independent experiments. AF3, AlphaFold 3; ISG15, Interferon Stimulated Gene 15; USP16, ubiquitin-specific peptidase 16.

We identified USP16 as an ISG15 crossreactive deubiquitinating enzyme (DUB) that can process proISG15 and ISGylated substrates (16). Although a structure of the USP16–ISG15 complex remains to be determined, AF3 predicts that ISG15 interacts with USP16 at both N- and C-terminal domains (Fig. 5*A*). The prediction is consistent with the experimental structure of the homologous USP18–ISG15 complex from mouse (Protein Data Bank [PDB] entry: 5CHV) (34). When we compare the AF3 model of USP16 binding to ISG15 with our crystal structures of ISG15 bound to VHH_ISG15-A_ or VHH_ISG15-B_, we would predict that only VHH_ISG15-A_ would exhibit mutually exclusive binding because of a steric clash (Fig. 5*A*).

To explore whether ISG15-specific nanobodies affect USP16-mediated deISGylation *in vitro*, we incubated a twofold molar excess of each VHH with the precursor of human ISG15 (pro-ISG15_1–165_). We then assessed cleavage by inclusion of recombinant human USP16 catalytic domain (amino acids 196–823) (16). The addition of VHH_ISG15-A_ abolished proISG15 processing by USP16, whereas VHH_ISG15-B_ did not (Fig. 5*B*). To further investigate this, we prepared cell lysates from IFN-β-stimulated HAP1 cells deficient in USP18 (USP18KO) and incubated these lysates with the anti-ISG15 nanobodies. Consistent with our *in vitro* findings, VHH_ISG15-A_ completely abolished the deconjugation of endogenous ISGylated substrates by purified recombinant USP16CD, whereas VHH_ISG15-B_ had no effect (Fig. 5*C*).

## Discussion

We developed two novel nanobodies, VHH_ISG15-A_ and VHH_ISG15-B_, that target distinct epitopes on human ISG15 and showed their potential as tools to investigate the biological roles of ISG15. These nanobodies address a critical need for specific, high-affinity reagents to study ISG15 and its associated pathways. By leveraging the unique properties of nanobodies, including their small size, stability, and high-yield production, we have expanded the experimental toolkit for probing ISG15 biology.

The distinct recognition patterns of VHH_ISG15-A_ and VHH_ISG15-B_, which target the C- and N-terminal domains of ISG15, respectively, provide complementary tools. The ability of the nanobodies to bind simultaneously to ISG15 suggests potential applications in sandwich-based detection methods; for example, a nanobody-based ELISA (35, 36). The nanomolar binding affinities, particularly of VHH_ISG15-B,_ show their robust binding properties.

The ability of both nanobodies to efficiently recover ISGylated proteins from cellular lysates with minimal background contamination highlights their potential for future proteome-wide studies. Limitations associated with traditional antibody-based approaches would thus be circumvented. VHH_ISG15-A_, in particular, proved compatible with immunoblotting under reducing conditions, providing a useful tool for the specific detection of ISG15 in complex samples. Our proteomic analysis shows that the anti-ISG15 reagents detect shared as well as unique targets. Accessibility of the ISG15 moiety to the nanobody or antibody reagents in a diverse array of possible ISGylated substrates may account for the recovery of substrates unique to a given retrieval agent. It underscores the utility of having multiple anti-ISG15 reagents for proteomic analyses.

Functionally, we observed that VHH_ISG15-A_, but not VHH_ISG15-B_, inhibits USP16-mediated deISGylation. This finding is consistent with structural predictions that VHH_ISG15-A_ binding to the C-terminal domain of ISG15 induces steric hindrance at the USP16 binding interface. This selective inhibition highlights the potential of VHH_ISG15-A_ to modulate ISG15 processing and provides a unique opportunity to dissect the biological consequences of ISGylation and its reversal. Such mechanistic insights may facilitate the development of therapeutic strategies targeting dysregulated ISG15 pathways in diseases, such as cancer, neurodegenerative disorders, and inflammation. These tools might also prove valuable for diagnostic purposes; the nanobodies' small size and high specificity make them particularly suitable for such applications (37).

There are limitations to our study. First, while we demonstrated high specificity and affinity for human ISG15, the lack of crossreactivity with mouse ISG15 constrains the use of these nanobodies in preclinical mouse models. Given the more limited sequence similarity of ISG15 with Ub or with other Ub-like modifiers, such as SUMO, crossreactions of VHH_ISG15-A_ and VHH_ISG15-B_ are not expected, although not verified experimentally. One of the hallmarks of antibody–antigen recognition is its specificity for the cognate antigen. A high degree of sequence similarity is typically required for crossreactions to occur. This stands in contrast to the promiscuous nature of some of the Ub-specific proteases and activity-based probes. Future efforts could focus on generating mouse-specific reagents or on the use of human ISG15 transgenic mice. While VHH_ISG15-A_ inhibited USP16 activity *in vitro*, further studies are needed to explore the potential of these nanobodies for intracellular expression to manipulate ISG15 pathways in living cells. For example, understanding whether and how nanobodies affect ISGylation dynamics and downstream signaling pathways in IFN-stimulated cells could provide deeper insights into ISG15-related mechanisms.

Our work also raises questions about the differential roles of the N-terminal and C-terminal domains of ISG15. The distinct binding specificities of VHH_ISG15-A_ and VHH_ISG15-B_ as shown also by proteomic analysis offer an opportunity to investigate these domains independently. For instance, future studies could use these nanobodies to probe how each domain contributes to ISG15’s biological functions, such as protein–protein interactions, secretion, or its extracellular cytokine-like activities.

In conclusion, the VHH_ISG15-A_ and VHH_ISG15-B_ nanobodies not only enable the precise detection and isolation of ISGylated substrates but also provide a means to interrogate the functional consequences of ISG15 interactions and modifications. By applying these tools to diverse experimental systems, we anticipate new insights into the complex roles of ISG15 in health and disease.

## Experimental procedures

### Preparation of phage display nanobody library and screening

Methods for preparation and panning of the phage display library were followed as described (38, 39). In brief, an alpaca was immunized with 250 μg of purified recombinant human ISG15-SBP-His protein, with four injections administered at 2-week intervals. Peripheral blood mononuclear cells were isolated, and total RNA was extracted using an RNeasy mini kit (Qiagen). cDNAs were synthesized using Superscript III First Strand Synthesis System (Thermo Fisher). VHH sequences were PCR amplified from the cDNA and cloned into a phagemid vector through restriction enzyme digestion and ligation. The resulting nanobody library was electroporated into TG1 *E. coli* cells (Agilent). The library was then infected with M13KO7 helper phage (NEB) to generate phage particles, which were subsequently harvested by centrifugation and PEG–NaCl precipitation.

Selection of ISG15-specific VHHs was accomplished through two rounds of panning against immobilized human ISG15-SBP-His protein on Dynabeads MyOne Streptavidin T1 magnetic beads (Thermo Fisher). The initial round used 20 μg of immobilized protein. Bound phage was eluted using 0.2 M glycine (pH 2.2) for 10 min, followed by neutralization with 1 M Tris-HCl (pH 9.1). The eluted phage was amplified in log-phase *E. coli* ER2738 (NEB) to generate a first-round library. A second round of selection was performed using this enriched library against 2 μg of immobilized protein. Final screening by ELISA, using plate-immobilized human ISG15-SBP-His protein, led to the identification of 20 unique nanobody sequences.

### Cell culture

HeLa (catalog no.: ATCC CCL-2) cells were cultured under standard conditions in Dulbecco’s modified Eagle’s medium (Gibco) supplemented with 10% fetal calf serum (GeminiBio) and 1% penicillin–streptomycin at 37 °C and 5% CO_2_. Chronic myelogenous leukemia–derived HAP1 USP18KO cells (Horizon; catalog no.: HZGHC000492C011) (26) were cultured in Iscove's modified Dulbecco's medium (Sigma–Aldrich) supplemented with 10% fetal calf serum (GeminiBio), 1% penicillin–streptomycin at 37 °C, and 5% CO_2_.

HeLa cells were seeded to achieve 50% to 60% confluence and transfected using TransIT-LT1 Transfection Reagent (Mirus Bio) following the manufacturer’s instructions. HeLa and HAP1 USP18KO cells were treated with 1000 U/ml of recombinant human IFN-β (PeproTech; catalog no.: 300-02BC for the indicated times.

### DNA constructs

proISG15 (2-165) (catalog no.: 110762; Addgene) (40), pET15b-mISG15(GG) (catalog no.: 12448; Addgene) (41), pET30b-5M SrtA (catalog no.: 51140; Addgene) (42), Tetra-FLAG–tagged human ISG15 (pEGFP-C1 vector) (16) have been previously described. Human ISG15 (1-157)-SBP-His and human UbcH8-SBP-His were cloned into the pET30b vector. Nanobody VHH_ISG15-A_ and VHH_ISG15-B_ sequences were synthesized by IDT and subcloned into a pHEN6 vector downstream of an N-terminal peIB sequence and equipped with a C-terminal LPETGG sortase motif-HA tag–His6 tag. Mutations (human ISG15 C78S, human ISG15 V74D, and VHH_ISG15-B_-F98D) were introduced by site-directed mutagenesis.

### Protein expression and purification

#### ProISG15, mISG15, human ISG15-SBP-His, human UbcH8-SBP-His, and sortase 5M

*E. coli* BL21 (DE3) cells containing expression plasmids were grown in terrific broth to an absorbance of 0.4 to 0.8 at 600 nm, induced with 1 mM IPTG, and incubated at 25 °C for overnight at 220 rpm. Cells were pelleted, resuspended in lysis buffer (50 mM Tris–HCl, pH 7.5, 150 mM NaCl, cOmplete EDTA-free Protease Inhibitor, and DNAse I), and lysed with an Avastin Emulsiflex C3 homogenizer. Lysate was cleared by centrifugation, and the soluble fraction was run over pre-equilibrated nickel–nitrilotriacetic acid (Ni–NTA) beads. Beads were washed with lysis buffer containing 10 mM imidazole and eluted in lysis buffer with 250 mM imidazole. Eluates were further purified *via* SEC using a Superdex S75 16/600 column (Cytiva) and stored in Tris-buffered saline (TBS) buffer (50 mM Tris–HCl [pH 7.5], 150 mM NaCl) at −80 °C.

#### VHH_ISG15-A_ and VHH_ISG15-B_

*E. coli* WK6 cells containing expression plasmids were grown in terrific broth at an absorbance of 0.8 at 600 nm, induced with 1 mM IPTG, and incubated at 30 °C for overnight at 220 rpm. For 1 l culture broth, cells were pelleted, resuspended in 15 ml of TES buffer (0.2 M Tris–HCl [pH 8.0], 0.65 mM EDTA, and 0.5 M sucrose), and incubated for 1 h at 4 °C. Osmotic shock was triggered by adding 75 ml water, followed by overnight incubation at 4 °C. Following centrifugation, the supernatant containing VHHs was run over pre-equilibrated Ni–NTA beads. The procedures of Ni–NTA purification and SEC were performed as aforementioned.

Protein sequence of VHH_ISG15-A_ and VHH_ISG15-B_ used in this study. The motif for sortase reaction is highlighted in *green*, HA tag is highlighted in *red*.

>VHH_ISG15-A_-HA-His protein sequence

QLQLVETGGGLVQAGGSLRLSCAASGRTFAGLSVKWFRQPPGAEREWVAHITSTGSSTHYADSVKGRFTISRDYDRNMVYLQMSSLKPEDTAVYYCHASTLWPEKHQDYWGQGIQVTVSSGGLPETGGSSYPYDVPDYAGGSSHHHHHH

>VHH_ISG15-B_-HA-His protein sequence

QVQLVETGGGLVEPGGSLRLSCTVSGINFSSQFMAWFRQGPGKQREWVAIDAGEENVGYLDLVEGRFTISRDTARNTLYLQMNSLKPEDTAVYYCNIFPVLPDSRWGQGTQVTVSSGGLPETGGSSYPYDVPDYAGGSSHHHHHH

Purification of recombinant USP16 has been previously described (43).

Protein concentrations were determined by UV spectroscopy using extinction coefficients at 280 nm based on their amino acid composition.

### Sortase reaction

To generate C-terminally biotin-tagged VHHs, 50 μM VHHs were mixed with 1 μM sortase A (5M), 500 μM GGG-Biotin in TBS buffer containing 10 mM CaCl_2_, and the reaction mixture was incubated at 4 °C overnight. Unreacted components and Sortase A 5M were removed using Ni–NTA beads. The flowthrough was further purified by SEC on PD10 columns (Cytiva; catalog no.: 17085101).

To generate untagged human ISG15 (C78S) protein for BLI experiments, 50 μM VHHs were mixed with 1 μM sortase A (5 M), 500 μM Tri–glycine (Sigma–Aldrich; catalog no.: G1377) in TBS buffer containing 10 mM CaCl_2_, the reaction mixture was incubated at 4 °C overnight. Unreacted components and Sortase A 5M were removed using Ni–NTA beads. The flowthrough was further purified by SEC on PD10 columns.

### Electrophoresis and immunoblots

Samples were resolved by SDS-PAGE using TRIS–glycine–SDS running buffer. Gels were stained with InstantBlue Coomassie Protein Stain (ISB1L) (abcam; catalog no.: ab119211); or gels were silver stained with Pierce Silver Stain Kit (ThermoFisher Scientific; catalog no.: 24612).

For immunoblots, the gel was transferred to a nitrocellulose membrane (Bio-Rad, 0.2 μm, catalog no.: 1620112) at 300 mA for 3 h. The membranes were blocked in 5% milk in 1× PBS. VHHs were added at 1 μg/ml in blocking buffer for 1 h at room temperature. The membrane was then washed in PBS with Tween three times, and the secondary antibody was added and incubated for 30 min at room temperature. Following this, the membrane was washed three times in PBS with Tween, and the signal was developed using Pierce ECL Western Blotting Substrate (Thermo Fisher; catalog no.: 32106), and the membranes were imaged using Bio-Rad ChemiDoc MP.

The following antibodies were used (dilutions indicated): ISG15 Recombinant Rabbit Monoclonal Antibody (7H29L24) (ThermoFisher Scientific; catalog no.: 703131, Research Resource Identifier [RRID]: AB_2784562, 1:1000 dilution), HRP-conjugated streptavidin (ThermoFisher Scientific; catalog no.: N100, 1:10,000 dilution), HA Tag Monoclonal Antibody (2-2.2.14), HRP (ThermoFisher Scientific; catalog no.: 26183-HRP, RRID: AB_2533056, 1:10,000) dilution, Goat anti-Rabbit IgG (H+L) Secondary Antibody, HRP (ThermoFisher Scientific; catalog no.: 31460; RRID: AB_228341, 1:20,000 dilution), Goat anti-Mouse IgG (H+L) Secondary Antibody, and HRP (ThermoFisher Scientific; catalog no.: 31430, RRID: AB_228307, 1:20,000 dilution).

### SEC binding experiments

Indicated molar ratios of VHH:(human/mouse) ISG15 at 20 μM concentration in 500 μl TBS were mixed and incubated at room temperature for 20 min. The mixture (450 μl) was applied to a Superdex 75 10/300 column (Cytiva; catalog no.: 17517401) at a flow rate of 0.5 ml/min, collecting 0.5 ml elution fractions. Samples were analyzed on 15% SDS-PAGE.

### BLI analysis

BLI assays to evaluate the binding kinetics between VHH_ISG15-A_–VHH_ISG15-B_ and human ISG15 (C78S) were conducted on an Octet RED384 (Sartorius) using Octet Ni–NTA Biosensors (Sartorius; catalog no.: 18-5101). Biosensor tips were prehydrated in BLI buffer (50 mM Tris–HCl [pH 7.5], 150 mM NaCl, 0.05% Tween-20, and 0.1% [w/v] bovine serum albumin) for 10 min, loaded with C-terminally His-tagged VHH_ISG15-A_ or VHH_ISG15-B_, and equilibrated in BLI buffer for baseline stabilization. Association was measured by exposing biosensors to varying concentrations of ISG15 (C78S) for 250 s, with concentrations ranging from 15 to 600 nM for VHH_ISG15-A_ and 1.88 to 60 nM for VHH_ISG15-B_. Dissociation was monitored in BLI buffer for 200 to 300 s. Data were reference-subtracted and fitted to either a 1:1 binding model (VHH_ISG15-A_) or a mass transport model (VHH_ISG15-B_) to calculate the equilibrium dissociation constant (*K*_*d*_) using Octet Analysis software. The mass transport model was selected for VHH_ISG15-B_ as it provided the best visual fit to the data, accounting for potential diffusion limitations because of the high *k*_on_ value, with improved χ^2^ (0.0008) and *R*^2^ (0.9929) values. Results were visualized in GraphPad Prism (GraphPad Software, Inc).

### VHHs enrich ISGylated substrates from cell lysates

IPs were performed as described (16) with the following modifications. HAP1 USP18KO cells were stimulated with 1000 U/ml IFN-β (PeproTech; catalog no.: 300-02BC) for 48 h to induce ISGylation. Cells were pelleted, resuspended in lysis buffer (20 mM Hepes [pH 8.0], 150 mM NaCl, 0.2% NP-40, 10% glycerol, 5 mM *N*-ethylmaleimide, and phosphatase and protease inhibitor cocktails), and incubated on ice for 30 min. Lysate was cleared by centrifugation and subjected to IP using 20 μg of C-terminally biotin-tagged VHH_ISG15-A_ or VHH_ISG15-B_ and 30 μl of Pierce NeutrAvidin Agarose slurry (ThermoFisher Scientific; catalog no.: 29200) for 16 h at 4 °C. Beads were washed four times with lysis buffer. Immune complexes were eluted with 4X Laemmli sample buffer. One-tenth of the eluates was used for immunoblotting with the indicated antibodies. The remaining eluate was prepared for analysis by MS.

### ISG15 interactomics analysis by LC–MS/MS using the VHH_ISG15-A_ and VHH_ISG15-B_ nanobodies

IP experiments were carried out with modifications based on previously established protocols (26). HAP1 USP18 KO cells were treated with recombinant human IFN-α2b (PBL Assay Science; catalog no.: 11105-1; final concentration: 500 U/ml) for 48 h. After treatment, cells (2.5 × 10^7^ per condition) were lysed in a buffer containing 20 mM Hepes (pH 8.0), 150 mM NaCl, 0.2% NP-40, 10% glycerol, 5 mM *N-*ethylmaleimide, and phosphatase and protease inhibitor cocktails. Lysates were subjected to IP using 5 μg of anti-ISG15 antibody (Thermo Fisher; catalog no.: 703131) with 25 μl protein G Sepharose (Invitrogen; catalog no.: 15920-10) or 10 μg of HA-tagged VHH_ISG15-A_ or VHH_ISG15-B_ with 100 μl anti-HA agarose beads (Pierce; catalog no.: 26182) for nanobody pulldown. Incubations were performed at 4 °C for 16 h. Beads were then washed four times with the lysis buffer, and immune complexes were eluted using 2X Laemmli buffer.

A portion (10%) of the eluates was analyzed *via* immunoblotting with the indicated antibodies. The remaining eluates were processed for MS analysis using suspension trap technology. Proteins were first reduced with 200 mM DTT in 0.1 M Tris (pH 7.8) and subsequently alkylated in the dark using 200 mM iodoacetamide in water. Acidification was achieved with 12% phosphoric acid before loading the samples onto suspension trap mini columns (ProtiFi; C02-midi). The columns were washed with 90% methanol in 100 mM triethylammonium bicarbonate and centrifuged at 4000*g*. Protein digestion was carried out overnight at room temperature using trypsin at a 1:100 (enzyme:protein) ratio. Resulting peptides were dried and reconstituted in buffer A (98% Milli-Q water, 2% acetonitrile, and 0.1% trifluoroacetic acid).

Peptides (200 ng per sample) were analyzed using an Evosep One liquid chromatography system coupled to a timsTOF HT mass spectrometer (Bruker) equipped with a 1.5 μm, 8 cm × 150 μm analytical column (Evosep). The separation was carried out using the 60 samples-per-day method with solvent A (0.1% formic acid in water) and solvent B (acetonitrile with 0.1% formic acid). The column was maintained at 40 °C. Data were acquired in parallel accumulation serial fragmentation–data-independent acquisition mode across a mass range of 100 to 1700 *m/z* and an ion mobility range from 1/*K*_0_ = 1.30 to 0.85 Vs/cm^2^, using equal accumulation and ramp times of 100 ms in the dual TIMS analyzer. Each acquisition cycle included eight parallel accumulation serial fragmentation ramps, with 25 Da windows covering the 475 to 1000 *m/z* range and two-thirds nonoverlapping ion mobility windows. Collision energy was modulated based on ion mobility, decreasing from 59 eV at 1/*K*_0_ = 1.6 Vs/cm^2^ to 20 eV at 1/*K*_0_ = 0.6 Vs/cm^2^.

Raw data were processed using DIA-NN (v1.8.1) with default settings and the "match between runs" feature enabled. Searches were performed against the UniProt *Homo sapiens* database including isoforms (20,412 entries; downloaded on December 13, 2023). Output data (report.pg_matrix) were further analyzed using Perseus software (v2.0.10.0) (https://maxquant.net/perseus/), and volcano plots and Manhattan distances were generated using VolcaNoseR (https://huygens.science.uva.nl/VolcaNoseR/).

### ProISG15 cleavage assays

Recombinant human pro-ISG15 protein was diluted in EMBO lysis buffer (50 mM Tris–HCl [pH 7.4], 150 mM NaCl, 2 mM EDTA, 0.5% NP-40, and 8 mM Tris(2-carboxyethyl)phosphine hydrochloride) to a final concentration of 4 μM, preincubated with 8 μM VHH_ISG15-A_, VHH_ISG15-B_, or both at room temperature for 20 min, and incubated with 0.5 μM of the recombinant USP16 catalytic domain at 37 °C for the indicated times. The reaction was stopped by boiling with LDS sample buffer.

### DeISGylation assays in cell lysates

HAP1 USP18KO cells were stimulated with 1000 U/ml IFN-β for 48 h to induce ISGylation. Cell pellets were lysed in EMBO lysis buffer (50 mM Tris–HCl [pH 7.4], 150 mM NaCl, 2 mM EDTA, 0.5% NP-40, 8 mM Tris(2-carboxyethyl)phosphine hydrochloride). Cell lysates (40 μg) were preincubated with 8 μM VHH_ISG15-A_, VHH_ISG15-B_, or both at room temperature for 20 min, then incubated with recombinant USP16 CDWT at a final concentration of 5 μM at 37 °C for 2 h. The reaction was stopped by boiling with LDS sample buffer.

### NMR analysis of binding epitope

^15^N-labeled ISG15 was produced by expressing human ISG15(1-157)-His6 in M9 minimal media containing ^15^NH_4_Cl. Cells were grown at 37 °C, 220 rpm until an absorbance at 600 nm reached 0.4, then induced with 1 mM IPTG, and grown at 30 °C, 220 rpm for 24 h. NMR experiments were performed using 0.5 mM ^15^N-labeled ISG15, 110% molar equivalent of VHHs (when present), in NMR buffer (50 mM Na_2_HPO_4_, pH 7.0, 100 mM NaCl, 10% D_2_O). ^15^N-transverse relaxation optimized spectroscopy heteronuclear single quantum coherence data were collected at 25 °C on a Bruker Advance II 600 MHz spectrometer equipped with a Prodigy cryogenic probe. Data were processed using NMRPipe, and analysis and plots were generated using CARA software (http://cara.nmr.ch/doku.php/Home). Combined 1H and 15N chemical shift changes of estimated NMR peak shifts were calculated using the formula: sqrt((ΔHcs)ˆ2 + (ΔNcs/5.0)ˆ2).

### Crystallization

Purified VHH_ISG15-A_ was incubated with recombinant human ISG15 at a 1:1 M ratio for 30 min at room temperature. The resulting mixtures were then applied to a Superdex S200 10/300 column pre-equilibrated with TBS buffer (20 mM Tris–HCl, pH 7.4, 150 mM NaCl). The heterodimeric complex was collected, and VHH binding was verified using SDS-PAGE followed by Coomassie blue staining. The complex was subsequently concentrated to 20 mg/ml in TBS buffer using a 10 kDa cutoff protein concentrator.

The purified complex of ISG15 and VHH_ISG15-A_ was crystallized at a concentration of 20 mg/ml by vapor diffusion in 0.3 μl sitting drops with a protein to reservoir ratio of 1:1. Crystals appeared within 3 days and continued to grow over the next 3 weeks in 25% (w/v) PEG 3350, 0.1 M trisodium citrate (pH 5.6), and 0.2 M ammonium sulfate at 18 °C. The rod-shaped crystals were mounted on nylon loop, cryoprotected with reservoir solution supplemented with 20% (v/v) ethylene glycol, and flash frozen in liquid nitrogen.

### Data collection and processing

Diffraction data were collected at the FMX beamline at NSLS II (BNL) on an Eiger X 16M detector (44). The data were indexed, integrated, and scaled using XDS (https://xds.mr.mpg.de/) (45). The structure was solved by molecular replacement with PHASER-MR (https://phenix-online.org/) (46) using ISG15 (PDB entry: 3PSE (47) and an AF predicted VHH as models. Model building and refinement was performed using Coot (https://www2.mrc-lmb.cam.ac.uk/personal/pemsley/coot/) (48) and phenix.refine (https://phenix-online.org/) (49). Data collection and refinement statistics are summarized in Table 1.

### Data availability

Coordinates and structure factor for the VHH_ISG15-A_–ISG15 complex are available at the PDB as entry 9NN9.

## Supporting information

This article contains supporting information.

## Conflict of interest

The authors declare that they have no conflicts of interest with the contents of this article.
