# Supplementary material for: Identification and characterization of nanobodies specific for the human ubiquitin–like ISG15 protein
Source: J Biol Chem. 2025 Aug 6;301(9):110564. doi: 10.1016/j.jbc.2025.110564 (PMC12406269; doi:10.1016/j.jbc.2025.110564)
Supplement: Supplementary Figures [file mmc1.docx]

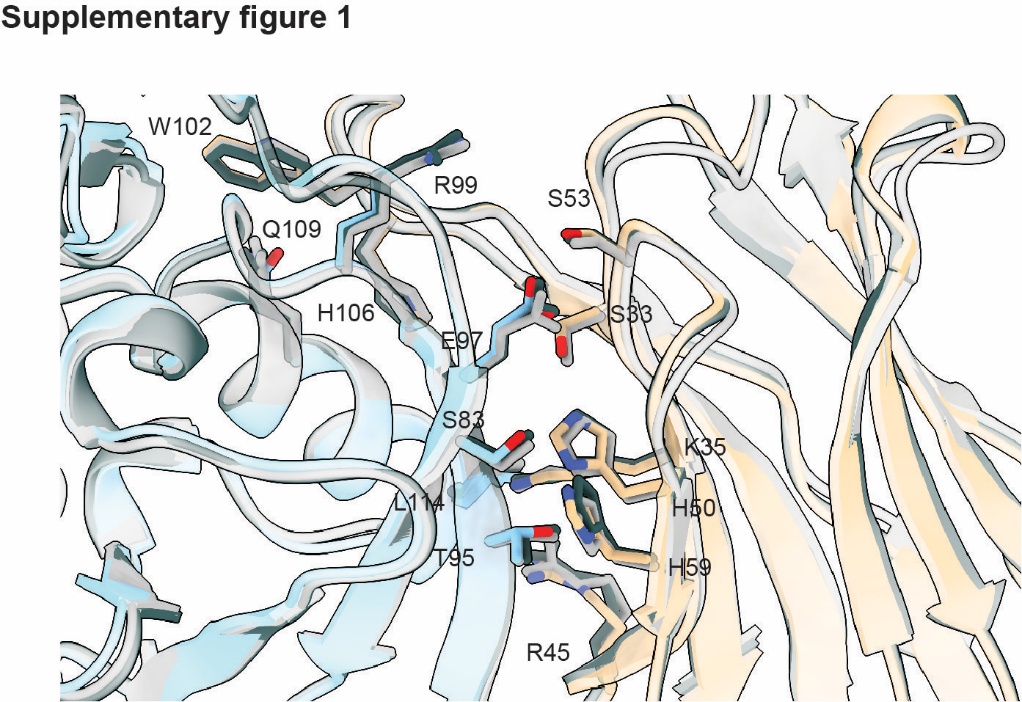


**Supplementary Figure S1**

Superposition of the three independent ISG15-VHH_ISG15-A_ complexes in the asymmetric unit. Same close-up of the binding interface as in Fig. 3D. The interfaces are virtually identical.


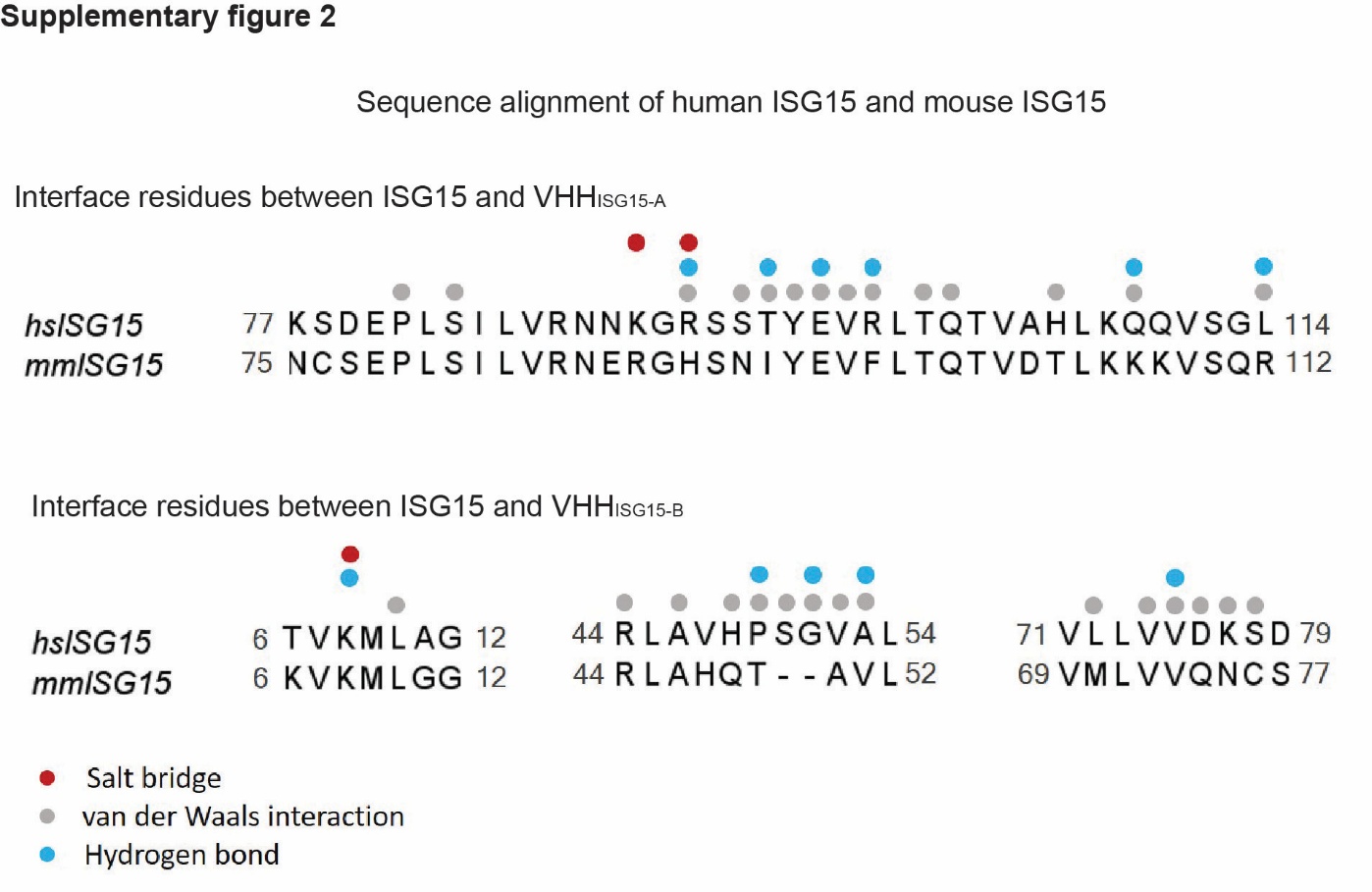


**Supplementary Figure S2**

Sequence alignment of human and murine ISG15, focused on the interaction regions with nanobodies VHH_ISG15-A_ and VHH_ISG15-B_, respectively. The binding interface is only modestly conserved, explaining the specificity of the nanobodies for human ISG15.
